# Evaluating Tartary Buckwheat Genotypes with High Callus Induction Rates and the Transcriptomic Profiling during Callus Formation

**DOI:** 10.3390/plants12213663

**Published:** 2023-10-24

**Authors:** Haixia Zhao, Xin Li, Xin Xiao, Tao Wang, Lisong Liu, Chenglei Li, Huala Wu, Zhi Shan, Qi Wu

**Affiliations:** College of Life Science, Sichuan Agricultural University, No. 46, Xinkang Road, Ya’an 625014, China; zhaohaixia@sicau.edu.cn (H.Z.); 18080188217@163.com (X.L.); 15228145102@163.com (X.X.); biowangtao@sicau.edu.cn (T.W.); liu657913897@163.com (L.L.); lichenglei@sicau.edu.cn (C.L.); hualawu@sicau.edu.cn (H.W.); mesopig@hotmail.com (Z.S.)

**Keywords:** Tartary buckwheat, callus, genotype, transcriptome

## Abstract

Due to their complex genotypes, low in vitro regeneration rates, and difficulty in obtaining transgenic plants, studies concerning basic biological research and molecular breeding in Tartary buckwheat (TB) are greatly limited. In this study, the hypocotyls of 60 genotypes of TB (TBC1~60) were used as explants. Of these, TBC14 was selected due to a high callus induction rate of 97.78% under dark and a proliferation coefficient (PC) of 28.2 when cultured on MS medium supplemented with 2.0 mg/L of 2,4-D and 1.5 mg/L of 6-BA. Subsequently, the samples of the calli obtained from TBC14 were collected at 0, 10, 20, and 30 d, and their transcriptomes were sequenced where identified. GO enrichment led to the detection of the most significant active gene set, which was the DNA binding transcription factor activity. The DEGs related to the pathways concerning metabolism, the biosynthesis of secondary metabolites, and hormone signal transduction were the most enriched in the KEGG database. The sets of MYB, AP2/ERF, and bHLH TFs exhibited the highest number of DEGs. Using this enrichment analysis, 421 genes encoding TFs, 47 auxin- and cytokinin-related genes, and 6 signal transduction-associated genes were screened that may play significant roles in callus formation (CF) in TB. Furthermore, *FtPinG0008123200.01* (bZIP), a key gene promoting CF, was screened in terms of the weighted gene co-expression network associated with the various stages of CF. Our study not only provides valuable information about the molecular mechanism of CF but also reveals new genes involved in this process.

## 1. Introduction

Tartary buckwheat (*Fagopyrum tataricum* (L.) Gaertn.) is a widely cultivated grain crop, used as a food source due to its balanced nutrition profile and used in medicine owing to its rich flavonoid content [1]. In recent years, Tartary buckwheat (TB) has attracted significant attention as a raw material for green foods, but its applicability is hampered due to its poor taste and low yield. Therefore, the improvement of TB varieties has become the focus of concerted research groups. Traditional methods like interspecific hybridization used to acquire desirable traits in TB are disadvantageous due to a long breeding cycle and being cumbersome. Hence, utilizing the recently developed molecular biology techniques for improving TB has become a popular method of choice. With the complete sequencing of the TB reference genome, the mining of key genes encoding the enzymes involved in the flavone biosynthesis pathways, the molecular mechanisms involved in their transcriptional regulation, and the functional identification of genes encoding stress-resistance-related transcription factors (TFs) have become a hotspot in this field of research [2,3,4]. However, an immature in vitro regeneration and genetic transformation system in TB has greatly hindered the progress. Callus, a crucial stage in plant regeneration [5], is also a significant target for achieving a breakthrough in regeneration in TB. Rumyantseva et al. [6,7] successfully established a long-term culture of morphogenic calli in Tartary buckwheat. Kostyukova and Rumyantseva [8] discovered a correlation between the formation of an embryogenic callus and specific competent cells located in the procambial and subepidermal layers of immature embryo cotyledons in Tartary buckwheat. Han et al. [9] and Kwon et al. [10] have successfully developed protocols for somatic embryogenesis in Tartary and common buckwheat from hypocotyls. These protocols involve the induction of callus formation followed by the regeneration of plantlets, utilizing a complex combination of growth regulators. Genotypes are critical factors that influence callus formation (CF) in TB. Different buckwheat spp. demonstrated varying efficiencies of callus induction (CI), with the common buckwheat generally exhibiting a better CI efficiency than TB [11]. Therefore, extensive screening is needed to identify genotypes with high-quality regenerative capabilities. Simultaneously, appropriate hormone combinations and conditions concerning in vitro culture are critical for promoting regeneration in TB. An efficient in vitro regeneration protocol has been reported for common buckwheat. This involved the addition of 0.5 mg/L 2,4-D and 0.2 mg/L 6-BA with sucrose, which demonstrated the highest induction of somatic embryogenesis (SE) from the cotyledons or the hypocotyls used as explants [12]. Jiujiang TB outperformed Wensha common buckwheat in the regeneration ability, with a maximum CI of up to 93.3% achieved by the addition of 3 mg/L 2,4-D and 0.5 mg/L 6-BA [13]. Furthermore, the regeneration ability of Yuanzi was superior to that of Xichang TB. In particular, the hypocotyls as explants exhibited a higher level of plantlet generation via SE than the cotyledons of TB. The most suitable medium for CI was the basal MS medium supplemented with 2 mg/L 2,4-D and 1 mg/L KT, which demonstrated a CI of up to 98.96% [11].

In addition, understanding the molecular mechanisms underlying CF is also helpful in improving the regeneration efficiency. According to previous reports, the plant hormone signaling pathway is involved in callus formation, and the differential genes of callus formation in *Arabidopsis thaliana* [14], *Dimocarpus longan Lour* [15], and *Picea balfouriana* [16] are enriched in the plant hormone signaling pathway. The hormone-signaling pathways and TFs are crucial in regulating the CF in plants; TFs such as WOX, LBD, LEC1, LEC2, FUS3, and ABI3 play significant roles in CI and somatic embryogenesis in *Arabidopsis* [5,17,18,19,20]. The expression of *BBM* and *WUS2* led to higher transformation frequencies in various recalcitrant inbred lines of maize [21]. Expression of a fusion protein comprising the wheat GRF4 and its cofactor GIF1 significantly enhanced the efficiency and rate of regeneration in wheat, triticale, and rice while enhancing the number of transformable genotypes in wheat [22]. Furthermore, the mechanisms behind CF and differentiation vary across plant species, and unraveling them requires specifically targeted studies.

In this study, the hypocotyls of 60 genotypes of TB (TBC1~60) were used as explants to screen them for high rates of CI, optimize their culture conditions regarding the light intensity and hormone ratio, and conduct a preliminary analysis of the CF mechanism using transcriptomic sequencing. The observations made may serve as a foundation for establishing an efficient regeneration and genetic transformation system in TB.

## 2. Results

### 2.1. Screening of the Genotypes of TB for a High Rate of CI

**Figure 1:**
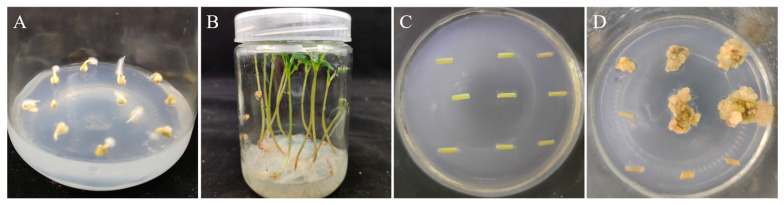
The process of CI in TB. (**A**): 3 d aseptic seedlings; (**B**): 12 d aseptic seedlings; (**C**): 0 d of callus induction; (**D**): 45 d of callus induction.

The genotypes with the highest rates of embryogenic CI were selected from the 60 genotypes of TB (Supplementary Table S1). The results of the seedling-germination assay showed that the germination rate was >90% on the third day of culture, which grew vigorously up to the 12th day. The callus morphology was assertable after the hypocotyl was removed and the calli were transferred to the callus induction medium (CIM) and cultured for 45 d under dark conditions (Figure 1).

**Figure 2:**
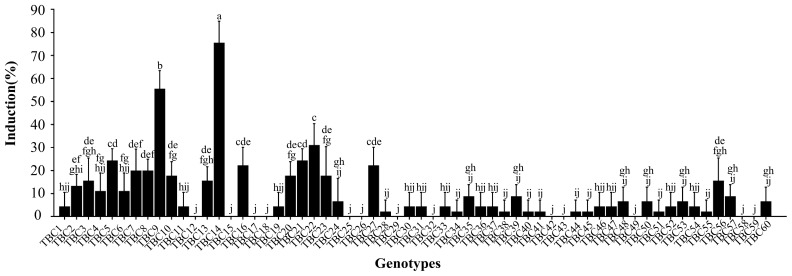
Statistics of CI rates in the selected 60 genotypes of TB. Note: The error bars represent the standard errors. Significant differences are indicated by lowercase letters: a, b, c, etc.; (*p* < 0.05).

Significant differences in the CI rates were observed among the selected genotypes. Of these, only two genotypes—TBC9 (55.56%) and TBC14 (75.56%)—demonstrated CI rates > 50%; in seven genotypes—TBC5 (24.44%), TBC16 (22.22%), TBC21 (24.44%), TBC22 (31.11%), TBC27 (22.22%), TBC7 (20%), and TBC8 (20%)—the CI rates were 20~50%; and the CI rates of the remaining genotypes were <20% (Figure 2).

**Figure 3:**
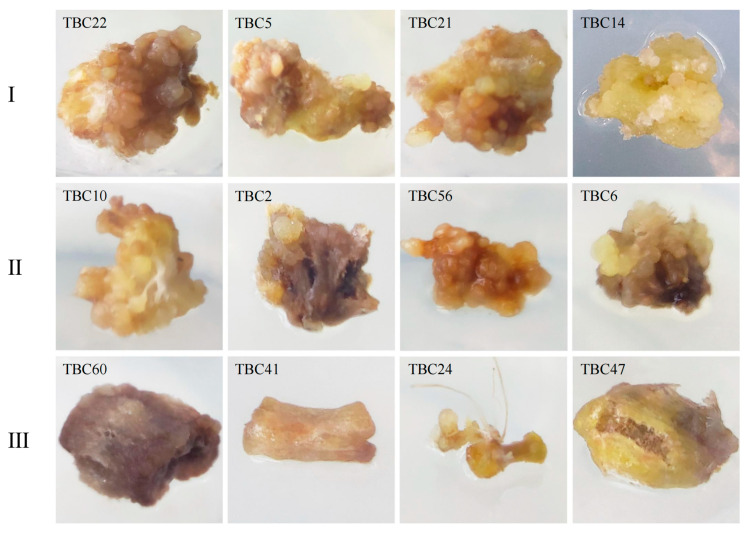
Phenotypes of the calli obtained from the TB genotypes with different CI rates after culture on a CIM for 45 d. Type I: genotypes with callus induction rate greater than 20%; Type II: genotype with callus induction rate of 10~20%; Type III: genotype with callus induction rate less than 10%.

Similarly, significant differences in the morphology of the calli obtained from the various genotypes with different CI rates were also observed. The calli from TBC60, TBC41, TBC24, and TBC47 with CI rates of 0~10% were deep brown and the granular tissues on the surface of the hypocotyl explants were reduced after expansion. However, the expansion in TBC24 was not as obvious as the others, but adventitious roots were produced. The calli induced in TBC10, TBC24, TBC56, and TBC6 with CI rates of 10~20% significantly expanded in size, with enhanced granular tissues, and an increase in the brown area. The surface of the calli in the genotypes with a CI rate >20% was covered with granular tissues, while the brown area was reduced and lighter in color (Figure 3).

Finally, the seven genotypes (TBC5, TBC9, TBC14, TBC16, TBC21, TBC22, TBC27) with the highest CI rates were selected for the subsequent screening step.

### 2.2. Effects of Light Intensity on the CI Rates in the Seven TB Genotypes

**Figure 4:**
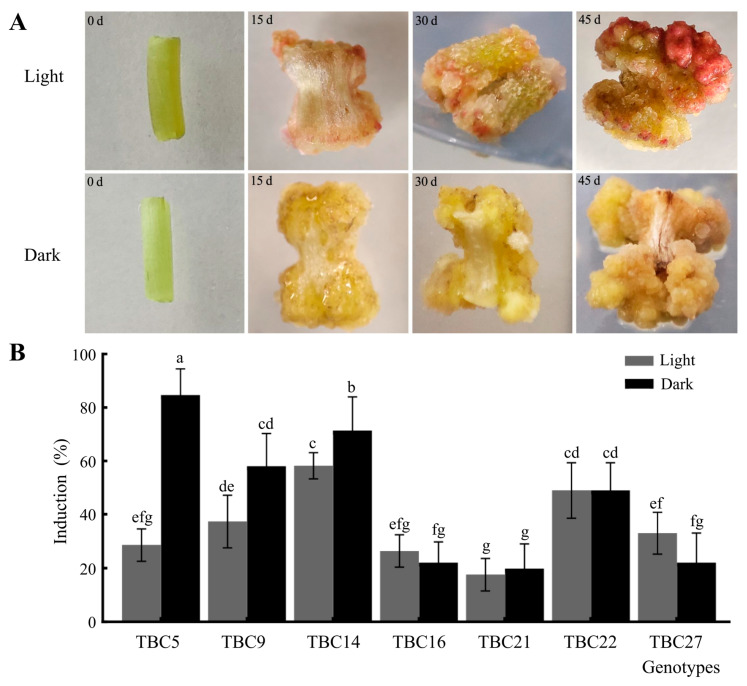
Effects of the two conditions of light exposure on the CI in the seven genotypes of TB selected after the initial screening. (**A**): Callus morphology cultured under the two lighting conditions. (**B**): Callus induction rate of 7 genotypes under different light conditions. Note: The error bars represent the standard errors. Significant differences are indicated by lowercase letters: a, b, c et al. (*p* < 0.05).

The seven genotypes selected after the primary screening were grown under different in vitro culture conditions (dark/light photoperiod of 16 h/8 h, light intensity of 2.0 klx, 25 °C) and dark culture (25 °C). Visible differences were observed in the color of the calli induced under the two different conditions of light exposure. Under illumination, CF was accompanied by the accumulation of anthocyanins resulting in a red phenotype, while those induced under the dark were light yellow (Figure 4A). The CI rates in the seven genotypes were higher in the secondary screening than those obtained in the initial screening and were varied under the dual conditions of light exposure. These rates in TBC5, TBC9, and TBC14 under dark conditions were significantly higher than under light conditions, while the rates in the other four genotypes were not significantly different under the dual conditions of light exposure. The CI rates in TBC5 and TBC14 in the dark were not only significantly higher than those in the light but also higher than those of the other six genotypes (Figure 4B). Therefore, TBC5 and TBC14 were selected for the third screening step.

### 2.3. Effect of Hormone Ratio on the CI Rates in Two Genotypes of TB, TBC5 and TBC14

To determine the highest CI rates regarding the optimal hormone ratio, the calli of TBC5 and TBC14 were transferred to a medium with varying hormone concentrations and cultured under dark conditions. After 45 d, the phenotypes were observed, the CI rates were calculated, and the proliferation coefficients (PCs) were estimated.

**Figure 5:**
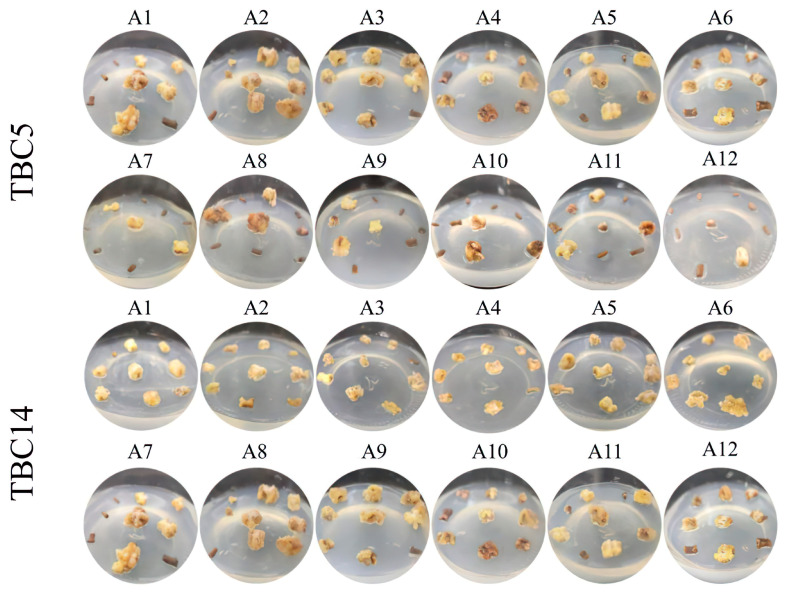
CI in TBC5 and TBC14 by the different combinations of hormones. Note: A1–A12 medium with various hormone ratios (Supplementary Table S2).

The observations of the callus morphology revealed that when 6-BA was at 0.5 mg/L, the hypocotyls of TBC5 expanded significantly on the A1~A3 media with the increasing concentrations of auxin and an increase in granular tissues, while the calli grown on the A4 medium demonstrated a significant browning. The hypocotyls of TBC14 conspicuously expanded on the A1~A4 media and had a color indicating healthy growth, without an obvious difference. At 1.5 mg/L of 6-BA, certain surfaces of the hypocotyls of TBC5 were vitrified with an evident browning after 45 d of growth on the A5~A8 media and the calli induced on A8 were dark brown. The calli induced on the A5 and A6 media from the hypocotyls of TBC14 had a color indicating good health with a pronounced swelling. However, the calli formed on the A7 and A8 media not only demonstrated severe browning but also white vitrified tissues on their surfaces. At 2.5 mg/L of 6-BA, the calli formed by the hypocotyls of TBC5 on the A9 medium were smaller, but they were light yellow indicating good health, while those on the A10~A12 media were severely vitrified and partially brown. The surfaces of the calli obtained from TBC14 on the A9~A12 media were vitrified and with fewer granular tissues (Figure 5).

**Figure 6:**
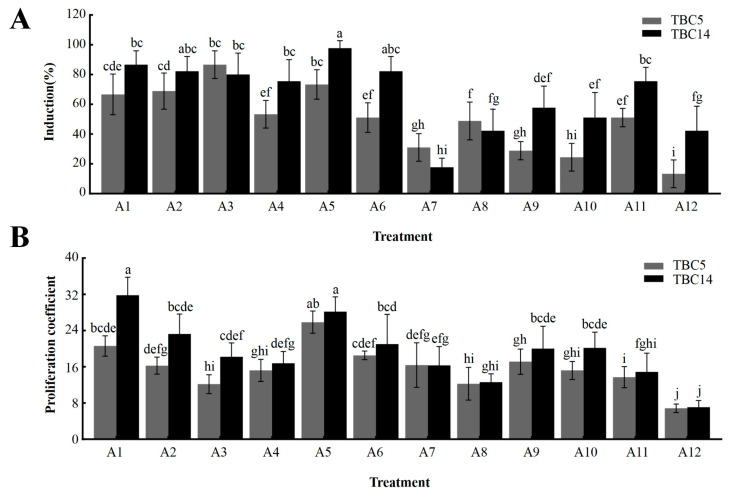
Effects of the different concentrations of hormones on CI in TBC5 and TBC14. (**A**): Callus induction rate after 45 days of TBC5 and TBC14; (**B**): proliferation coefficient after 45 days of TBC5 and TBC14. Note: The error bars represent the standard errors. Significant differences are indicated by lowercase letters: a, b, c, etc.; (*p* < 0.05).

A comparison of the CI rates and the PCs of the two genotypes indicated that on the A1 and A2 media, TBC5 had a slightly greater CI rate than TBC14, but this was not significantly different. Similarly, on the A3, A7, and A8 media, TBC5 had a slightly higher CI rate than TBC14, but this was not significantly different. The CI rate of TBC14 was higher than that of TBC5 on the other media and significantly higher than those of all the remaining genotypes on A5 (Figure 6A). At similar concentrations of 6-BA, the PC was inversely correlated with the concentration of 2,4-D. The PC of TBC14 on the A1 and A5 media were significantly higher than those on the others (Figure 6B).

In summary, TBC14 was identified as the genotype with the best CI rate, especially on the A5 medium (2.0 mg/L 2, 4-D, and 1.5 mg/L 6-BA), which was 97.78% and with a PC of 28.2. Additionally, the morphological characteristics of the induced calli proclaimed excellent health.

### 2.4. Transcriptomic Analysis of the Calli Obtained from TBC14

**Figure 7:**
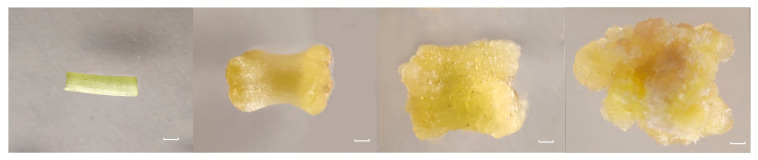
Morphology of the calli obtained at 0, 10, 20, and 30 d after induction in TBC14. Note: the ruler represents 1 mm.

The hypocotyls of TBC14 were used as explants to induce CF on an optimized CIM containing 2.0 mg/L 2, 4-D and 1.5 mg/L 6-BA. Based on the morphological changes occurring during the process of CF, the sampling time points were 0 d (callus 0), 10 d (callus 10), 20 d (callus 20), and 30 d (callus 30); 0~10 d, 10~20 d, and 20~30 d were defined as the early, middle, and late periods of CF (Figure 7). To obtain a complete mRNA expression profile during the entire process of CF, cDNA libraries were constructed from the calli samples collected in triplicate at the aforementioned four-time points.

### 2.5. Global Transcriptomic Changes

After the screening for and exclusion of adaptor sequences and low-quality reads, ~765 million reads with high-quality scores were identified. More than 85% of these clean reads had a minimum quality score of Q30 with a GC content > 45%. The quality of the sequenced reads was ascertained to be appropriate for the subsequent analysis (Supplementary Table S4).

### 2.6. Differential Gene Expression (DEG) Analysis

**Figure 8:**
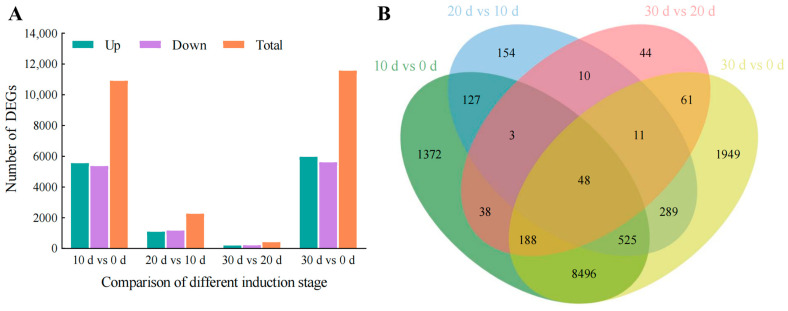
The profile of the DEGs identified during the process of CF in TB. (**A**): Statistics of DEGs; (**B**): Venn diagram of DEGs.

To compare the differences in the gene expression profiles at specific stages of CF, the read numbers were first normalized to the FPKM value (Supplementary Table S5). During the entire process of CF, 11,567 genes were identified to exhibit a significant differential expression, with 5964 being upregulated and 5603 downregulated. The analysis of the DEGs at different stages of CF revealed that 5545 genes were upregulated and 5354 were downregulated during the early stage of CF (10 d over 0 d), while the expression of 48 genes was altered during all the three periods (Figure 8). These results suggest that there are significant differences in gene expression at different stages of callus development and that many genes are needed to ensure complete differentiation.

### 2.7. GO Analysis of the DEGs

**Figure 9:**
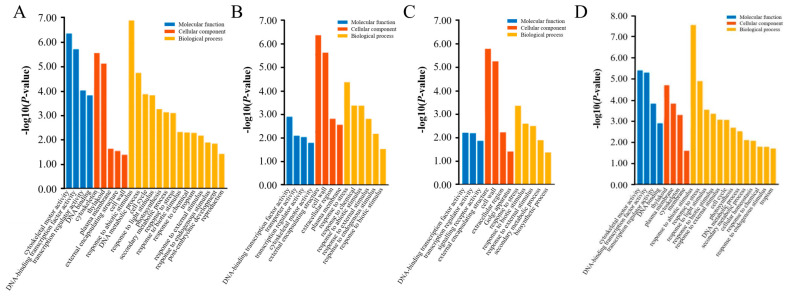
GO enrichment analysis of the DEGs identified during the process of CF in TB. (**A**): 10 d vs. 0 d; (**B**): 20 d vs. 10 d; (**C**): 30 d vs. 20 d; (**D**): 30 d vs. 0 d. Note: the horizontal coordinates are −log_10_^(*p* value)^ processed, and entries are arranged in ascending order of *p* value.

To identify the biological functions of the DEGs identified during the different stages of CF, GO term enrichment analysis was conducted using the clusterProfiler tool. The results obtained showed that the DEGs in each period were mainly associated with the function of cytoskeletal motor activity, DNA-binding activity of TFs, and the activity of gene-transcriptional regulation; those mainly related to the cell organelles such as the cytoskeleton, plasma membrane, external encapsulation structure, and cell wall; and those concerning primary biological processes like responses to biotic or abiotic stimuli such as light and the secondary metabolic processes (Figure 9).

### 2.8. KEGG Analysis of the DEGs

**Figure 10:**
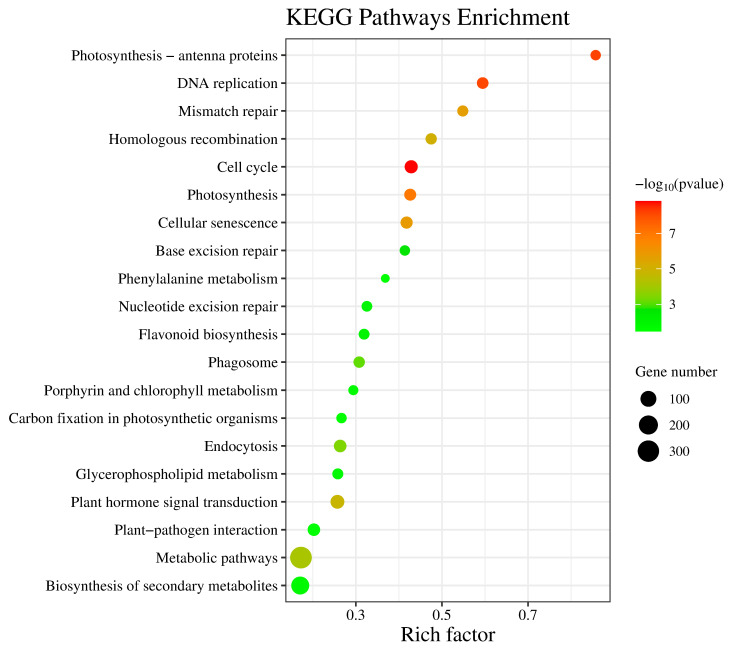
The bubble diagram representing the KEGG enrichment of DEGs at 10 d over 0 d. Note: The abscissa denotes the Rich factor that corresponds to the pathway, while the ordinate represents the name of the pathway. The hue of the dots represents the size of −log_10_^(*p* value)^. The bubble color indicates the *p* value. The size of the dots indicates how many differential genes are present in each pathway.

The early stage may be the critical period in CF since it revealed the highest number of DEGs of all three stages. Hence, KEGG analysis was performed to identify the metabolic pathways associated with these DEGs during this specific period. A total of 4624 DEGs were assigned to 285 KEGG pathways. Among these, the most significant pathways (*p* < 0.001) were identified to be related to the cell cycle (ko04110, 42 genes), photosynthesis–antenna proteins (ko00196, 12 genes), DNA replication (ko03030, 22 genes), and photosynthesis (ko00195, 26 genes). The most enriched of these pathways is photosynthesis–antenna proteins. Interestingly, the pathways that were associated with the highest number of DEGs concerned metabolism (ko01100, 312 genes), followed by those concerning the biosynthesis of secondary metabolites (ko01110, 161 genes), and finally those concerning signal transduction pathways of hormones (ko04075, 54 genes) (Figure 10). This indicates that CI encompasses the interplay of various metabolic processes and signal transduction pathways.

**Figure 11:**
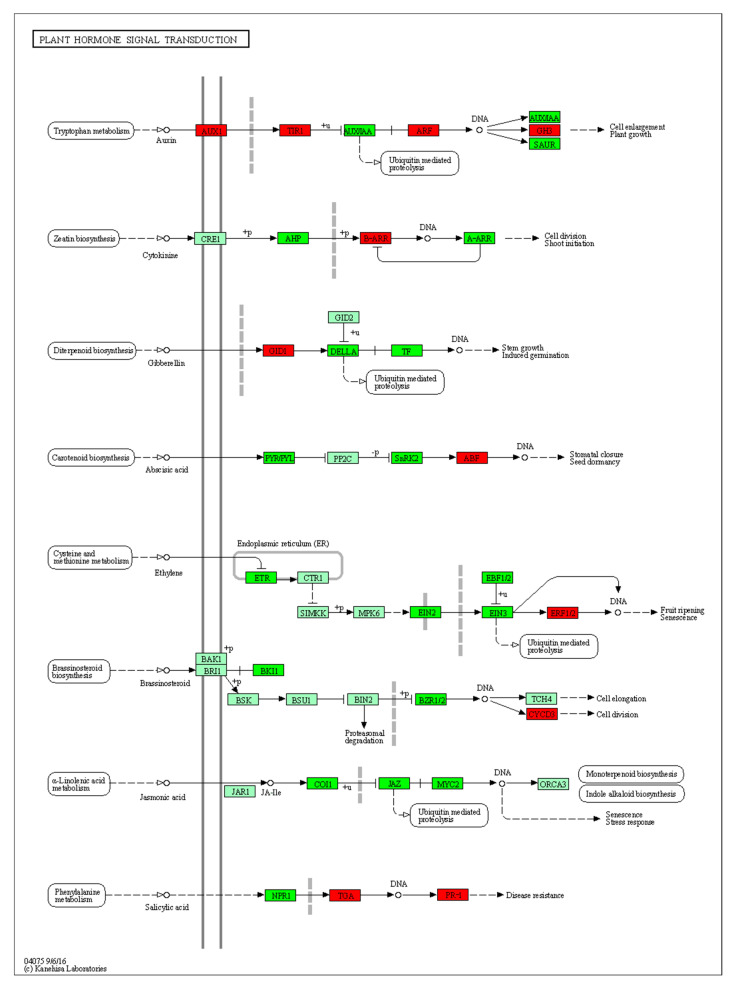
Enrichment of 10 d vs. 0 d DEGs in plant hormone signaling pathways. Note: The light green background in the figure indicates the genes existing in the KEGG pathway of the species genome; red background indicates up-regulated DEGs involved in the pathway, and dark green indicates down-regulated DEGs involved in the pathway.

Moreover, the plant hormone signaling transduction pathway exhibits a remarkable number of 54 DEGs, second only to metabolic pathways (312 DEGs) and secondary metabolite synthesis pathways (161 DEGs). The signal transduction pathways of plant hormones are illustrated in Figure 11. During the initial stages of TB callus formation, there is a selective enrichment of genes involved in various signaling pathways, including auxin, cytokinin, gibberellin, abscisic acid, brassinosteroid, salicylic acid, jasmonic acid and ethylene (Figure 11).

### 2.9. Validation of the DEGs by qRT-PCR

**Figure 12:**
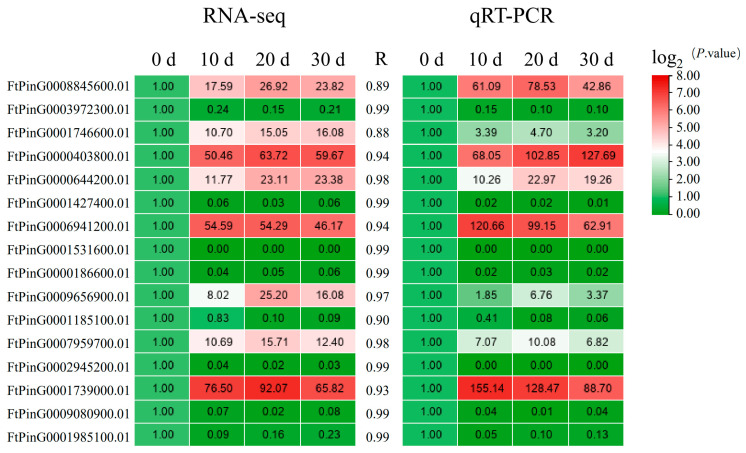
Validation of the profile of the DEGs using qRT-PCR. Note: R is the correlation coefficient. Columns and rows in the heatmap represent samples and genes, respectively. Sample names are shown below the heatmaps. The color bar explains the scale used to indicate the genes’ expression levels. The scale represents the logarithm of the FPKM value of gene expression.

To confirm the results of the transcriptomic studies, 16 genes with significant changes in their expression levels were selected from the DEGs for qRT-PCR analysis. A heat map was drawn based on the fold change in the gene expression levels over 0 d as the control. The correlation coefficient between the expression levels of the 16 DEGs and the results of the transcriptome sequencing for each period was calculated to be >0.88. Hence, the results of the transcriptome sequencing obtained in this study were deemed reliable and could be applied to the correlation analysis (Figure 12).

### 2.10. Analysis of Plant Hormone-Related Genes

**Figure 13:**
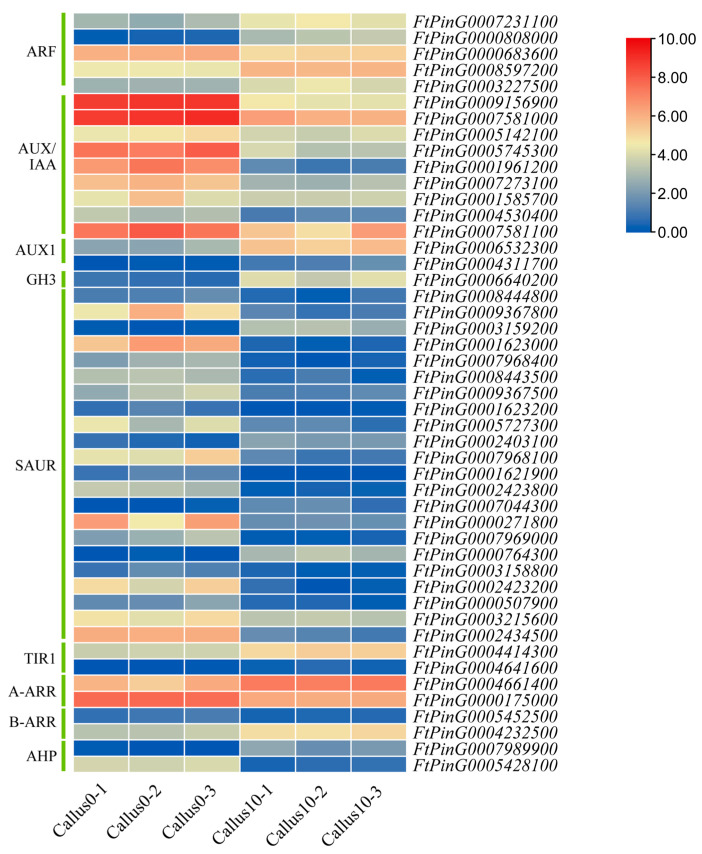
Heat map describing the auxin- and cytokinin-related DEGs at 10 d over 0 d. Columns and rows in the heatmap represent samples and genes, respectively. Samples names are shown below the heatmaps. The color bar explains the scale used to indicate the genes’ expression levels. The scale represents the logarithm of the FPKM value of gene expression.

Plant hormones are crucial to the formation of calli since 47 hormone-responsive genes were found to be differentially expressed in this process, which promoted the dedifferentiation of explants into calli. Several such genes were differentially expressed during CF: auxin-related genes *ARF*, *AUX1*, *GH3*, *TIR*, *AUX/IAA*, and *SAUR* in TB; cytokinin-related genes *AHP*, *A-ARR*, and *B-ARR*; gibberellin-related genes *DELLA*, *GID1*, and *PIF3*; jasmonate-related genes *JAZ*, *MYC2*, and *COI1*; salicylic acid-related genes *TGA*, *PR-1* (pathogenesis-related protein-1), and *NPR1*; brassinolide-related genes *BKI1*, *BZR1/2*, and *CYCD3*; abscisic acid-related genes *PYR/PYC*, *SnRK2*, and *ABF*; and finally, the ethylene-related genes *EIN*, *EIN2*, *ERF*, *ERF1/2*, and *ETR*. The changes in the expression of genes related to the plant hormones pathway were visualized using a heatmap, and those exhibiting similar expression patterns were categorized (Figure 13). It was observed that members within the same gene families displayed distinct expression patterns, indicating the intricate regulation of plant hormone networks during callus differentiation.

In tissue culture, the exogenous addition of auxins and cytokinins is pivotal to inducing dedifferentiation in the explants. In this study, on the 10th day of culture on CIM, a significant number of auxin- and cytokinin-related genes were found to be differentially expressed. Of these, the expression of *FtPing0003159200.01* (*SAUR*) was upregulated 105-fold and was the highest among all the auxin-related genes identified. Similarly, the expression of *FtPing0007989900.01* (*AHP*) was upregulated 135-fold and was the highest among all the cytokinin-related genes identified, suggesting that they may be a key regulatory factor in the initiation and maintenance of callus regeneration. Further experimental studies are needed to confirm the function of this gene.

### 2.11. Embryogenesis-Labeled Genes Involved in CI

**Figure 14:**
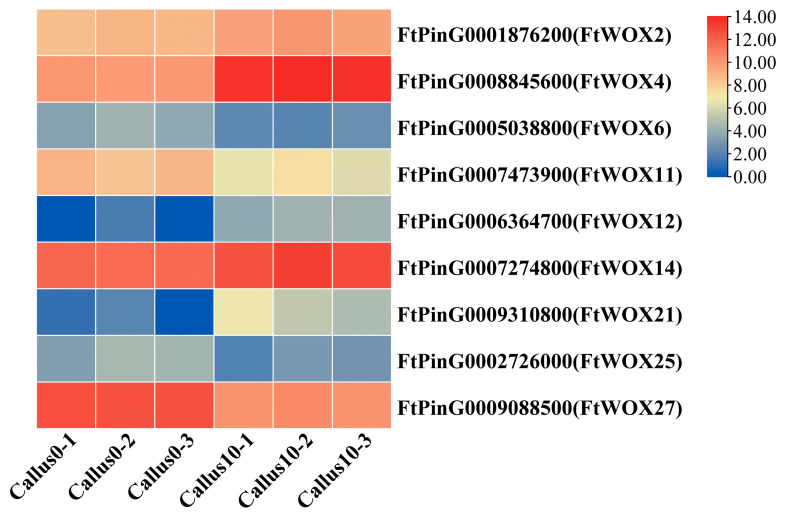
Heat map indicating the *WOX_S_* at 10 d over 0 d. Note: Columns and rows in the heatmap represent samples and genes, respectively. Sample names are shown below the heatmaps. The color bar explains the scale used to indicate the genes’ expression levels. The scale represents the logarithm of the FPKM value of gene expression.

The *WOX* gene family is widely acknowledged as prominent embryogenesis-labeled genes in plants, which have been reported to be involved in embryogenic callus induction. To gain insights into the characterization of these embryogenesis-labeled genes during Tartary buckwheat callus induction, we conducted a comparative analysis of their expression levels at 10 days compared to 0 days of induction. As a result, we identified nine differentially expressed genes belonging to the *WOX* gene family (Figure 14). Notably, genes such as *FtWOX4* (*FtPinG0008845600*) and *FtWOX14* (*FtPinG0007274800*) were found to be up-regulated during the callus initiation stage and maintained high expression levels throughout the later stages. Based on the embryogenesis-labeled genes analysis, our study indicates that the Tartary buckwheat callus exhibits characteristics associated with embryogenic development. This finding suggests that the callus has the potential for further differentiation and holds promise for future studies on callus differentiation in Tartary buckwheat.

### 2.12. Analysis of DEGs Encoding TFs

**Figure 15:**
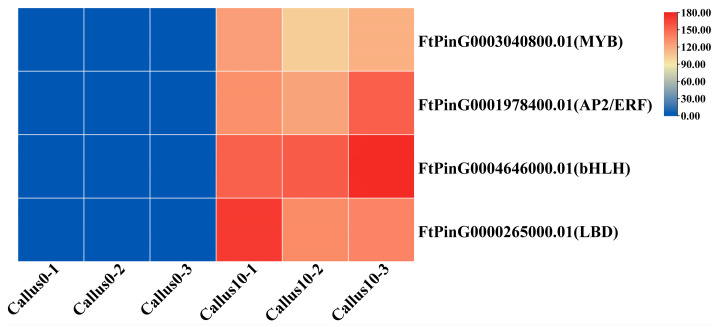
Heat map indicating the DEG encoding TFs with the highest up-regulation at 10 d over 0 d. Note: Columns and rows in the heatmap represent samples and genes, respectively. Sample names are shown below the heatmaps. The color bar explains the scale used to indicate the genes’ expression levels. The scale represents the multiples of gene expression.

The analysis of DEGs encoding TFs revealed that 421 TFs belonging to 40 TF families were involved in CF (Supplementary Table S6). Notably, the early stage demonstrated the highest number of such DEGs among the three periods of CF, suggesting that this stage is crucial during dedifferentiation. Of the 332 such DEGs identified, 163 were upregulated and 169 were downregulated. Among these genes, 39, 37, and 33 belonged to the TF families of MYB, AP2/ERF, and bHLH, respectively. In addition, 13 genes identified to be significant for plant regeneration belonging to the LBD family were also screened. Of the above-mentioned genes, the expression of *FtPinG0003040800.01* (MYB), *FtPinG0001978400.01* (AP2/ERF), *FtPinG0004646000.01* (bHLH), and *FtPinG0000265000.01* (LBD) was significantly upregulated on the 10th day of CI by 113-, 133-, 152-, and 145-fold when compared to the other members in their respective families (Figure 15). These results indicate that a substantial number of TFs synergistically regulate the dedifferentiation of explants during CF. The previous studies have demonstrated that these gene families exhibit differential expression patterns under stress conditions, thereby establishing a connection between stress and developmental pathways. All major TFs were found to be present in the mRNA transcriptome at each stage of development, with several TFs showing specific expression during distinct developmental periods.

### 2.13. Analysis of the Differential Gene Co-Expression Network

**Figure 16:**
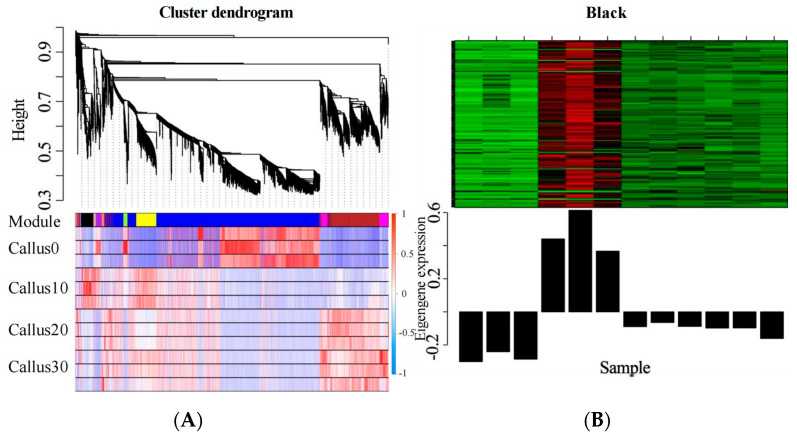
Construction of the co-expression network of the DEGs identified during CF in TB. (**A**): Gene clustering tree and module cutting; (**B**): Black module.

To study the gene regulatory network and determine the influential genes involved in the process of CF in TB, the DEGs were examined using the weighted gene co-expression network analysis (WGCNA) at various stages of CF. The DEGs were divided into a total of eight modules, of which the Black module-specific high-expression genes clustered to the 10th day of CI (Figure 16). Since the results previously obtained in this study suggested that the early stage may be significant to the whole CI process, the genes clustered in the Black module were selected for further analysis.

**Figure 17:**
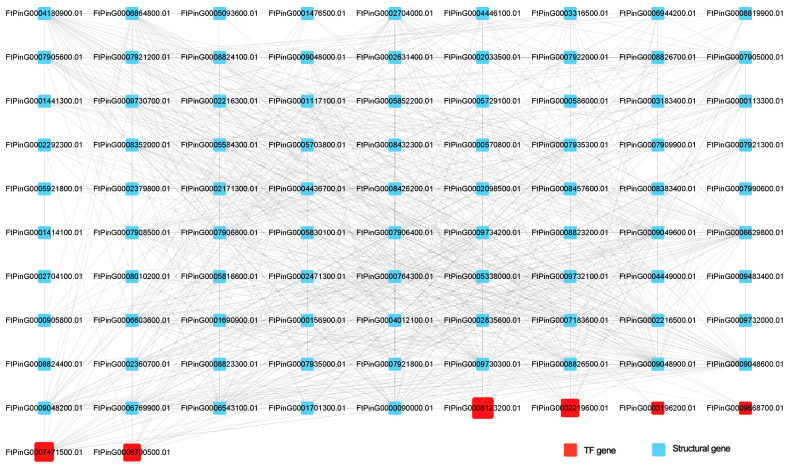
Gene co-expression network in the Black module.

There were 92 DEGs clustered in the Black module (Figure 17), including six TF-encoding genes that belonged to four different TF families—bZIP (*FtPinG0003196200.01*, *FtPinG0006700500.01*, and *FtPinG0008123200.01*), NAC (*FtPinG0007471500.01*), MYB (*FtPinG0002219600.01*), and Dof (*FtPinG0009668700.01*). *FtPing0008123200.01* demonstrated the highest connectivity of the Black module (degree = 22), which may be crucial for CF. However, the immature genetic transformation system of TB poses a challenge for further experimental validation of these genes’ functionality.

## 3. Discussion

### 3.1. The Factors Influencing CF in TB

The genotype of TB is a critical factor influencing its regeneration, and selecting the most suitable genotype is essential for establishing an effective regeneration system. As a key stage in indirect regeneration, the CI rate determines the regeneration efficiency in TB. Screening of genotypes for higher rates of CI is a prerequisite for establishing a high-efficiency regeneration system in TB. Among the 60 genotypes of TB selected in this study, TBC14 exhibited the highest CI rate, which could reach a maximum of 97.78% under the optimal hormonal ratio of 2.0 mg/L 2, 4-D and 1.5 mg/L 6-BA, and a maximum PC of 28.2.

Light is an important factor affecting CF and has different effects on divergent plant spp. For instance, date palm [23] was more responsive to CI in the dark, while the explants of rapeseed [24] demonstrated a lower rate of CI in the dark. In an earlier study, a higher CI rate was observed under light than dark conditions in TB [9]. In this study, seven genotypes of TB exhibited varying CI rates under different conditions of light exposure, which could explain the discrepancy in the results reported in the previous studies. However, further exploration is required to determine the underlying causes of these genotypic-based differences.

The formation of a callus in plants is subject to the influence of plant growth regulators, of which auxins and cytokinins have commonly been used in tissue culture for CI [25]. The auxin analogs used for CI in TB were mainly 2,4-D, IAA, and NAA; while the cytokinin analogs were mainly 6-BA, Kt, and TDZ. In previous studies, it was reported that 2,4-D was the sole inducer of CF in common buckwheat even without 6-BA. 2,4-D was identified to be a critical component of the CIM and high concentrations (3~4 mg/L) of 2,4-D were conducive for CI in TB [13]. The optimum hormonal composition of CIM was determined to be 3 mg/L 2,4-D and 1.5 mg/L 6-BA. In this study, 2,4-D was used at 2~4 mg/L and 6-BA at 0.5~1.5 mg/L; TBC5 demonstrated the highest CI rate at 3 mg/L of 2,4-D and 0.5 mg/L of 6-BA, while in TBC14, it was the highest at 2 mg/L of 2,4-D and 1.5 mg/L of 6-BA. It was observed that the different genotypes of TB have varying sensitivities to plant hormones. The concentration ranges of the hormones selected in this study were determined according to previous reports and a high rate of CF was obtained. The concentration of the hormones can be further refined in future studies to significantly improve the rate of CI.

### 3.2. Plant Hormone-Related Genes Play an Important Role in CF

Plant hormones serve as crucial factors in the initiation of callus formation. Additionally, genes involved in the hormone signal transduction pathway contribute significantly to the dedifferentiation process of explants. This study identified 41 auxin-related DEGs, which mainly included *ARF*, *AUX/IAA*, *AUX1*, *GH3*, *SAUR*, and *TIR1*. As an auxin response factor, *ARF* is known to regulate various cellular processes such as cell division, elongation, and differentiation [26]. *AUX1* functions as a vector for plant growth hormone transportation, facilitating the movement of growth hormones from the plasma membrane to the intracellular compartment. Furthermore, *AUX/IAA* forms a dimer with the growth hormone response factor ARF, leading to the inhibition of ARF gene expression [25]. The auxin receptor *TIR* is capable of binding to *AUX/IAA* to ubiquitinate and degrade it, and its ability to do so is positively correlated with the range of auxin concentrations [27]. *SAUR* plays a role in inhibiting the synthesis and transport of auxin. On the other hand, *GH3* forms a complex with IAA, rendering IAA temporarily nonfunctional. This interaction is involved in the homeostatic regulation of auxin levels. In the process of CF in Tartary buckwheat, the expression levels of genes *ARF*, *AUX1*, *GH3*, and *TIR1* were found to be upregulated. Conversely, the expression levels of *AUX/IAA* and *SAUR* genes were primarily downregulated. The findings suggest that the application of 2,4-D efficiently induces auxin signaling in explants, thereby promoting enhanced cell dedifferentiation.

Three cytokinin-related genes—*AHP*, *A-ARR*, and *B-ARR*—were identified in the study. *AHP* accepts the phosphate group of the cytokinin membrane receptor, and subsequently, they pass this phosphate group to downstream ARR class response regulators, enabling the regulation of downstream gene expression. *A-ARR* cytokinin responsive regulators can act as repressors to participate in cell expansion and plant development, while *B-ARR* class responsive regulators have the ability to positively regulate the expression of the *WUS* gene [28]. During the CF of Tartary buckwheat, it was observed that two genes from each of the three cytokinin-related gene groups showed differential expression patterns. These findings suggest that these genes have distinct roles in the overall process of callus formation and are likely work together to regulate and promote the production of callus tissue.

### 3.3. TF Play an Important Role in CF

TFs also play a significant role in regulating CF by controlling the expression of different functionally important genes. This analysis identified 421 TF-encoding genes, of which those belonging to the AP2/ERF, bHLH, and MYB TF families demonstrated the highest differential expression in the early stage of CF.

The TFs of one of the largest families, AP2/ERF are involved in a wide range of physiological processes in plants and some of its members were closely related to CF [29]. The members of the bHLH family can also promote CF and play a crucial role in somatic embryogenesis [30]. Certain members of the MYB family can also regulate CF [31]. Those of the LBD family are critical in plant regeneration and regulate CF in *Arabidopsis*, cotton, and strawberry [32,33,34]. The members of the Group I of the bZIP family play a crucial role in the early development of plants [35]. bZIP29 controls cell proliferation during early plant development, while bZIP59 interacts with LBD to promote CF [18,36].

In conclusion, the induction of callus in TB involves a wide variety of TFs belonging to multiple families, which constitute a regulatory network that promotes the transformation of explants into calli.

### 3.4. Embryogenesis-Labeled Genes Play Important Roles in CF

Several genes, such as *LEC1*, *BBM*, *WUS*, and *WOX*, have been identified as molecular markers of somatic embryogenesis (SE) [37]. In our study, we found differential expressions of *WOX* genes at different induction stages. In Arabidopsis, *WOX* genes have been identified as markers of cell fate during early embryogenesis [38]. During the regeneration of somatic embryos in grapes, the expression of *VvWOX3* and *VvWOX11* is activated specifically during the formation of torpedo embryos and cotyledons, thus playing a crucial role in regulating somatogenesis [39]. Similarly, in *Picea abies*, the *WOX2* and *WOX9* genes were found to be highly expressed during the early stage of somatic embryo development, indicating their potential collective role in conifer embryo patterning [40]. Moreover, *WOX* genes have been found to possess a diverse range of functions. For instance, *AtWOX5* is responsible for maintaining the activity of the root meristem [41], while *AtWOX2* serves as a vital factor in the formation of early embryos and cotyledons [42].

## 4. Materials and Methods

### 4.1. Plant Materials and Growth Conditions

A total of 60 Tartary buckwheat genotypes from different regions of the world were used in this study (Supplement Table S1). TB seeds were disinfected with 75% alcohol and 0.1% mercuric chloride, placed on 1/2 MS medium (0.7% agar, 2.37 g/L MS and 3% sucrose, pH = 5.8) and cultured in a light incubator for 10~12 days (d) (light culture for 16 h, dark culture for 8 h (h), light intensity of 2.0 klx, 25 °C), to obtain TB seedlings. Hypocotyls were cut into about 0.5 cm segments for callus induction.

### 4.2. Callus Induction and Genotype Screening

Hypocotyl segments from 60 different Tartary buckwheat genotypes were placed on MS medium (3.5 mg/L 2,4-D, 0.5 mg/L 6-BA) to induce callus. There were 5 replicates for each genotype and 9 explants for each replicate. The callus induction rate was determined after 45 days. Seven TB genotypes with the highest callus induction rate were selected for secondary screening, and the explants were placed in light culture (light/dark, 16 h/8 h, light intensity of 2.0 klx, 25 °C) and dark culture (25 °C) conditions to induce callus, during which they were subcultured every 15 days, callus induction rate was calculated after 45 days. Hypocotyl segments of the two TB genotypes with the highest callus induction rate were placed on MS medium with different hormone ratios (Supplement Table S2). During this period, they were subcultured every 15 days. Callus induction rate and proliferation coefficients were measured after 45 days.

Callus induction rate = number of callus/total number of explants

Proliferation coefficient = callus weight/initial explant weight after 45 days

### 4.3. Sample Preparation and Sequencing

According to the screening results, the hypocotyls of the genotypes with the highest callus induction rate of Tartary buckwheat were selected and callus induction was carried out under the optimal medium and light conditions for callus induction, and each subculture was conducted every 15 days. Take samples on the 0th, 10th, 20th, and 30th days of explant culture, and repeat three samples in each group (the samples are marked as callus_0-1, callus_0-2, callus_0-3, callus_10-1, callus_10-2, callus_10-3, callus_20-1, callus_20-2, callus_20-3, callus_30-1, callus_30-2, callus_30-3). The fresh weight of each sample is greater than 500 mg. After sampling, it was quickly frozen in liquid nitrogen and cryopreserved at ultra-low temperature, and sent to Shanghai Zhongke new life Biotechnology Co., Ltd. for eukaryotic participating transcriptome sequencing based on lllumina sequencing platform.

### 4.4. qRT-PCR

To verify the accuracy of the transcriptome sequencing data, sixteen differentially expressed genes were randomly selected and their primers were designed using the online software Primer3.0 https://bioinfo.ut.ee/primer3-0.4.0/ (7 February 2007), which were synthesized by Youkang Biotechnology Co., Ltd. (Chengdu, China) (Supplement Table S3). Take the RNA returned by APPLIED PROTEIN TECHNOLOGY (Shanghai, China) as the template and use PrimeScript™ RT reagent Kit to reverse transcribe it into cDNA. Tartary buckwheat *FtH3* (Genebank: HM628903) was used as an internal reference gene to detect the expression level of the corresponding gene. Reaction system (15 μL): 2 × TB Green Premix Ex Taq II 7.5 μL, cDNA 1 μL. Upstream primer (10 μM) 1.25 μL. Downstream primer (10 μM) 1.25 μL, ddH_2_O 4 μL. The amplification procedure is 95 °C, 45 s; (95 °C, 15 s; 58 °C, 45 s) 34 cycles. Three techniques were repeated for each sample, and the results were analyzed using 2^−ΔΔCt^ method.

### 4.5. Transcriptome Sequencing Data Analysis

To analyze the differential expression of genes between groups, DESeq2 http://bioconductor.org/packages/release/bioc/html/DESeq2.html (27 April 2007) was used to screen genes with (|log2(FC)| > 1, *Padj* < 0.05, *p* < 0.05). ClusterProfiler software v. 3.10.1. package was employed to study the enrichment of go energy and KEGG pathway. When *p* < 0.05, it was considered that there was significant enrichment of go or KEGG function. The differential genes were further screened (|log2(FC)| > 1.5, *Padj* < 0.00001, *p* < 0.00001), and the differential genes were annotated with transcription factors using PlantTFDB http://planttfdb.cbi.pku.edu.cn/ (26 October 2016). WGCNA (Weighted gene Co-expression Network Analysis) is carried out by using the baimaike cloud platform. The gene filtering threshold FPKM is set to 1, the Module fusion similarity threshold Fold is set to 0.5, the minModuleSize in the Module is 30, and the upper limit of the number of genes displayed in the interaction network ntop is 150 to build a co-expression network. Select the gene with weight > 0.4 and draw the network diagram with Cytoscape.

## 5. Conclusions

TBC14 was the genotype with the highest callus induction rate. The callus induction rate of 97.78% under dark and a proliferation coefficient (PC) of 28.2 when cultured on MS medium supplemented with 2.0 mg/L of 2,4-D and 1.5 mg/L of 6-BA.

Further analysis found that 421 genes encoding TFs, 47 auxin- and cytokinin-related genes, and 6 signal transduction-associated genes may play significant roles in callus formation (CF) in TB. Particularly, *FtPinG0008123200.01* (bZIP), a key gene promoting CF, was screened in terms of the weighted gene co-expression network associated with the various stages of CF.

## Data Availability

The datasets generated during and/or analyzed during the current study are available from the corresponding author on reasonable request.

## Supplementary Materials

The following supporting information can be downloaded at: https://www.mdpi.com/article/10.3390/plants12213663/s1, Table S1: The main distribution areas of 60 Tartary buckwheat genotypes; Table S2: Hormone combination of callus induction medium; Table S3: Primer sequence in this study; Table S4: The information about statistics of RNA-seq data; Table S5: Statistics table of the number of genes in different expression levels; Table S6: Analysis of differentially expressed transcription factor genes in callus at different developmental stages.

## Abbreviations

2.4-D	2,4-Dichlorophenoxyacetic acid
6-BA	6-Benzylaminopurine
*A-ARR*	type-A Response Regulator
*ABF*	ABRE binding factors
ABI3	abscisic acid-insensitive 3
*AHP*	Arabidopsis histidine-phosphotransfer
AP2	Apetala2
*ARF*	auxin response factor
*AUX*	auxin
*AUX1*	Auxin 1
*B-ARR*	type-B Response Regulator
*BBM*	BABY BOOM
bHLH	basic helix-loop-helix
*BKI1*	BRI1 KINASE INHIBITOR1
bZIP	basic leucine zipper, bZIP
*BZR1/2*	Brassinazole resistant 1/2
cDNA	complementary DNA
CF	callus formation
CI	callus induction
CIM	callus induction medium
*COI1*	CORONATINE-INSENSITIVE 1
*CYCD3*	Type-D cyclin 3
d	days
DEGs	differentially expressed genes
Dof	DNA binding with one finger
*EIN*	Ethylene-insensitive
*EIN2*	Ethylene-insensitive 2
*ERF*	ethylene responsive factor
*ERF1/2*	ethylene responsive factor 1/2
*ETR*	ethylene-response
FPKM	Fragments Per Kilobase Million
FUS3	FUSCA 3
*GH3*	Gretchen Hagen 3
*GID1*	GA-insensitive dwarf 1
GIF1	GRF-INTERACTING FACTOR 1
GO	Gene Ontology
GRF4	GROWTH-REGULATING FACTOR 4
h	hours
IAA	Indole-3-acetic acid
*JAZ*	JASMONATE ZIM
KEGG	Kyoto Encyclopedia of Genes and Genomes
KT	Kinetin
LBD	Lateral Organ Boundaries Domain
LEC1	LEAFY COTYLEDON1
LEC2	LEAFY COTYLEDON2
MS	Murashige and Skoog
MYB	v-myb avian myeloblastosis viral oncogene homolog
*MYC2*	Myelocytomatosis proteins 2
NAA	Naphthaleneacetic Acid
NAC	NAM, ATAF1/2, CUC1/2
*NPR1*	NONEXPRESSOR OF PATHOGENESIS-RELATED GENES 1
PCs	proliferation coefficients
*PIF3*	Phytochrome-interacting Factor 3
*PR-1*	pathogenesis-related protein-1
*PYC*	Pyruvate carboxylase
*PYR*	Pyrabatin Riesistance
qRT-PCR	quantitative real-time polymerase chain reaction
*SAUR*	Small auxin-up RNA
SE	somatic embryogenesis
*SnRK2*	sucrose non-fermenting-1-related protein kinase 2s
TB	Tartary buckwheat
TDZ	Thidiazuron
TFs	transcription factors
*TGA*	TGACG motif binding factor
*TIR*	Toll/Interleukin-1 Receptor
WGCNA	Weighted Gene Co-expression Network Analysis.
WOX	WUSCHEL-related homeobox
*WUS2*	WUSCHEL2
