# Supplementary material for: Evaluating Tartary Buckwheat Genotypes with High Callus Induction Rates and the Transcriptomic Profiling during Callus Formation
Source: Plants (Basel). 2023 Oct 24;12(21):3663. doi: 10.3390/plants12213663 (PMC10647830; doi:10.3390/plants12213663)
Supplement: Supplementary file 1 [file plants-12-03663-s001.zip › plants-2633661-supplementary.pdf]

Supplementary Table S1 The main distribution areas of 60 Tartary buckwheat genotypes.

Number	Region	Number	Region	Number	Region
TBC1	China	TBC21	China	TBC41	China
TBC2	China	TBC22	China	TBC42	China
TBC3	China	TBC23	China	TBC43	China
TBC4	China	TBC24	China	TBC44	China
TBC5	China	TBC25	China	TBC45	America
TBC6	China	TBC26	China	TBC46	Bhutan
TBC7	China	TBC27	China	TBC47	Bhutan
TBC8	China	TBC28	China	TBC48	America
TBC9	China	TBC29	China	TBC49	America
TBC10	China	TBC30	China	TBC50	America
TBC11	China	TBC31	China	TBC51	America
TBC12	China	TBC32	China	TBC52	America
TBC13	China	TBC33	China	TBC53	America
TBC14	China	TBC34	China	TBC54	America
TBC15	China	TBC35	China	TBC55	America
TBC16	China	TBC36	China	TBC56	China
TBC17	China	TBC37	China	TBC57	China
TBC18	China	TBC38	China	TBC58	China
TBC19	China	TBC39	China	TBC59	China
TBC20	China	TBC40	China	TBC60	China

Supplementary Table S2 Hormone combination of callus induction medium.

Medium number	2,4-D（mg/L）	6-BA（mg/L）
A1	2	0.5
A2	2.5	0.5
A3	3	0.5
A4	4	0.5
A5	2	1.5
A6	2.5	1.5
A7	3	1.5
A8	4	1.5
A9	2	2.5
A10	2.5	2.5

A11	3	2.5
A12	4	2.5

Supplementary Table S3 Primer sequence in this study.

Primer name	Upstream primer F (5’→3’)	Downstream primer R (5’→3’)	Purpose
Q-FtPinG0008845600.01	GACAGCAGCATTGATGCCTA	ATTCTCGTCCACTCCACCAC	qRT–PCR
Q-FtPinG0001531600.01	GTTGGAGCAACAATGGGAGT	TTTGTTGGTGGGAGTGATGA	qRT–PCR
Q-FtPinG0000403800.01	AGAGCCAAGGATTGCTTTCA	GATTGGGCTTGGATGAGTGT	qRT–PCR
Q-FtPinG0001739000.01	ACGGAGAAGGTAACGTGTGG	AACCTAAACCGTCCGATCCT	qRT–PCR
Q-FtPinG0000644200.01	TTCCGCCATGAATCTTCTCT	CCTTCGTTCTCATTCGCTTC	qRT–PCR
Q-FtPinG0001985100.01	AGTCATCACCAGCAGGCTCT	AACGCCTTACGCTTTGAAGA	qRT–PCR
Q-FtPinG0006941200.01	TCCGGCGAATATCTTAGGTG	TGTCGCTTTGTCAGTCTTGG	qRT–PCR
Q-FtPinG0003972300.01	CGAAACTGGAGACGGCTAAG	TTTACGTCGTCCTTCCCATC	qRT–PCR
Q-FtPinG0001746600.01	CATGCAATTGCCTAACATGG	ATTGGCATGAAATGGGTCAT	qRT–PCR
Q-FtPinG0001427400.01	ATGCGGCTGAGATCAGAGAT	ACTCCACGGTACTGGTCTGG	qRT–PCR
Q-FtPinG0009656900.01	CCGAGAAGCTACCTGGAGTG	TTTCTTTGCCTGCCGTAGTT	qRT–PCR
Q-FtPinG0000186600.01	CCGGGCTTGACAACAATACT	GTTGTTGCCCGGAAAGTAAA	qRT–PCR
Q-FtPinG0007959700.01	TACAAGGCCCAGAACCAAAC	AACCGCCAATTTTGTCTTTG	qRT–PCR

Q-FtPinG0001185100.01	CTGGATGAGAACGACCGATT	TTGCGACTCCCTAAGCAGAT	qRT–PCR
Q-FtPinG0009080900.01	TGGGAACTTTCGAGACTGCT	TCACCCATTCATCCAACTCA	qRT–PCR
Q-FtPinG0002945200.01	TTCATCGGAGGGATTATTGC	TACTGTTGTTGGGGTGACGA	qRT–PCR
Q-FtPinG0008845600.01	GACAGCAGCATTGATGCCTA	ATTCTCGTCCACTCCACCAC	qRT–PCR
Q-FtPinG0001531600.01	GTTGGAGCAACAATGGGAGT	TTTGTTGGTGGGAGTGATGA	qRT–PCR
Q-FtPinG0000403800.01	AGAGCCAAGGATTGCTTTCA	GATTGGGCTTGGATGAGTGT	qRT–PCR
Q-FtPinG0001739000.01	ACGGAGAAGGTAACGTGTGG	AACCTAAACCGTCCGATCCT	qRT–PCR

Supplementary Table S4 The information about statistics of rna-seq data.

Sample	Raw reads	Clean reads	Error(%)	Q20(%)	Q30(%)	GC(%)
Callus0-1	65361426	65354580	0.04	96.84	90.86	46.06
Callus0-2	60786140	60780104	0.04	96.8	90.74	45.88
Callus0-3	56582962	56577668	0.04	96.65	90.36	45.88
Callus10-1	59703810	59697526	0.04	96.73	90.51	45.86
Callus10-2	52349864	52344860	0.04	96.69	90.4	45.75
Callus10-3	89017898	89013350	0.05	95.35	86.72	45.3
Callus20-1	67772614	67765864	0.05	96.59	90.15	45.39
Callus20-2	68496814	68490858	0.05	96.2	89.11	45.2
Callus20-3	55117858	55112572	0.05	96.41	89.71	45.19
Callus30-1	63425786	63419540	0.05	96.54	90.02	45.47
Callus30-2	62751430	62745284	0.05	96.18	89.06	45.44
Callus30-3	64209404	64202454	0.04	96.74	90.51	45.4

Note: sample: sample name; raw reads: raw data of sequencing; Clean reads: the remaining data after filtering the original data; Clean bases: the number of clean reads is multiplied by the length of the sequencing sequence and converted into G; Q20 and Q30: the percentage of bases with base

mass fraction greater than 20 and 30 in the total base; GC: the percentage of the total number of bases G and C in the total number of bases.

Supplementary Table S5 Stat istics table of the number of genes in different expression levels.

Sample	0≤FPKM<1	1≤FPKM<5	5≤FPKM<10	10≤FPKM<30	30≤FPKM<50	50≤FPKM
Callus0-1	17922(48.3%)	5785(15.59%)	3484(9.39%)	5264(14.19%)	1624(4.38%)	3023(8.15%)
Callus0-2	17500(47.17%)	5907(15.92%)	3494(9.42%)	5408(14.58%)	1677(4.52%)	3116(8.4%)
Callus0-3	17571(47.36%)	5700(15.36%)	3586(9.67%)	5464(14.73%)	1743(4.7%)	3038(8.19%)
Callus10-1	16573(44.67%)	5753(15.51%)	3654(9.85%)	6131(16.52%)	1903(5.13%)	3088(8.32%)
Callus10-2	16439(44.31%)	5670(15.28%)	3645(9.82%)	6261(16.88%)	2011(5.42%)	3076(8.29%)
Callus10-3	16174(43.59%)	5669(15.28%)	3593(9.68%)	6502(17.52%)	2088(5.63%)	3076(8.29%)
Callus20-1	15723(42.38%)	5686(15.33%)	3690(9.95%)	6654(17.93%)	2172(5.85%)	3177(8.56%)
Callus20-2	15825(42.65%)	5636(15.19%)	3749(10.1%)	6647(17.92%)	2134(5.75%)	3111(8.38%)
Callus20-3	15835(42.68%)	5628(15.17%)	3690(9.95%)	6559(17.68%)	2199(5.93%)	3191(8.6%)
Callus30-1	15660(42.21%)	5651(15.23%)	3715(10.01%)	6776(18.26%)	2149(5.79%)	3151(8.49%)
Callus30-2	15672(42.24%)	5587(15.06%)	3757(10.13%)	6689(18.03%)	2205(5.94%)	3192(8.6%)
Callus30-3	16088(43.36%)	5656(15.24%)	3620(9.76%)	6460(17.41%)	2113(5.7%)	3165(8.53%)

Note: the percentage in brackets indicates the number of genes with relative abundance in different expression levels and its percentage in the total number of genes.

Supplementary Table S6 Analysis of differentially expressed transcription factor genes in callus at different developmental stages.

TF family	10 d vs 0 d		20 d vs 10 d		30 d vs 20 d		30 d vs 0 d	
	up	down	up	down	up	down	up	down
AP2/ERF	18	19	1		1		24	19
ARF	4	1					4	
ARR-B	1						1	1
B3	10	2					10	1
BES1	1						1	
bHLH	10	23	2	2			13	25
bZIP	9	12		1			6	14
C2H2	1	2		1			1	3
C3H	1	4		1			1	4
CAMTA		1					1	
CO-like		1						
CPP	1	1					1	
DBB	1	1					1	1
Dof	10	2				1	8	2
E2F/DP	2						1	1
EIL		2						1
G2-like	1	5					2	7
GATA	3	1					3	2
GeBP							1	
GRAS	4	6					6	6
GRF	1		1				2	
HB-other	1	1					2	
HD-ZIP	3	13					3	11
HSF	2	7		4			3	10
LBD	9	4	1				11	4
LSD	1	1					2	1
MADS	7	1					9	2
MYB	19	20	2	3	1		20	17
NAC	12	11	5	1			22	12
NF-Y	7	3	1				8	3
Nin-like	1			1				
RAV		1						1
SBP	1	1					2	
SRS	6						6	
TALE	1	6				1	2	5
TCP	2	3	1	1			6	3
Trihelix	2	3					1	5
WRKY	9	7	2				13	5
YABBY		1						1
ZF-HD	1	3						3
